# The importance of job characteristics in determining medical care-seeking in the Dutch working population, a longitudinal survey study

**DOI:** 10.1186/1472-6963-12-294

**Published:** 2012-08-31

**Authors:** Romy Steenbeek

**Affiliations:** 1 TNO Work and Health, PO Box 718,, 2130, AS, Hoofddorp, The Netherlands

## Abstract

**Background:**

The working population is ageing, which will increase the number of workers with chronic health complaints, and, as a consequence, the number of workers seeking health care. It is very important to understand factors that influence medical care-seeking in order to control the costs. I will investigate which work characteristics independently attribute to later care-seeking in order to find possibilities to prevent unnecessary or inefficient care-seeking.

**Methods:**

Data were collected in a longitudinal two-wave study (n = 2305 workers). The outcome measures were visits (yes/no and frequency) to a general practitioner (GP), a physical therapist, a medical specialist and/or a mental health professional. Multivariate regression analyses were carried out separately for men and women for workers with health complaints.

**Results:**

In the Dutch working population, personal, health, and work characteristics, but not sickness absence, were associated with later care-seeking. Work characteristics independently attributed to medical care-seeking but only for men and only for the frequency of visits to the GP. Women experience more health complaints and seek health care more often than men. For women, experiencing a work handicap (health complaints that impede work performance) was the only work characteristic associated with more care-seeking (GP). For men, work characteristics that led to less care-seeking were social support by colleagues (GP frequency), high levels of decision latitude (GP frequency) and high levels of social support by the supervisor (medical specialist). Other work characteristics led to more care-seeking: high levels of engagement (GP), full time work (GP frequency) and experiencing a work handicap (physical therapist).

**Conclusions:**

We can conclude that personal and health characteristics are most important when explaining medical care-seeking in the Dutch working population. Work characteristics independently attributed to medical care-seeking but only for men and only for the frequency of visits to the GP. The association between work characteristics and later medical care-seeking differed between health care providers and between men and women. If we aim at reducing health care costs for workers by preventing unnecessary or inefficient care, it is important to reduce the number of workers that report that health complaints impede their work performance. The supervisor could provide more social support, closely monitor workload in combination with work pressure and decision latitude, and when possible help to adjust working conditions. Health care providers could reduce medical costs by taking the work relatedness of health complaints into account and act accordingly, by decreasing the time to referral and waiting lists, and by providing appropriate care and avoiding unnecessary or harmful care.

## Background

Policymakers in western societies express great concern about increasing health care expenditures. High medical consumption is related to high rates of sickness absence [1-5] and both are costly to society. The working population is ageing, which will increase the numbers of workers with chronic health complaints, and, as a consequence, the number of workers seeking health care. Thus, it is very important to understand factors that influence medical care-seeking in order to find possibilities to prevent unnecessary or inefficient care-seeking.

Research on medical care-seeking behaviour is extensive. Studies on the working population not surprisingly show that health factors are most important when explaining care-seeking behaviour. Factors that are associated with more care-seeking include having a chronic disease or fatigue [6] and recurrent neck and/or low back pain (LBP) [7]. Severe (musculoskeletal) symptoms predict care-seeking [8].Studies on a selection of workers with health complaints show that the severity of LBP and significant disability predict [9] and the number of concurrent neck/shoulder symptoms is [10] is associated with more care-seeking.

Demographical factors are also found to influence care-seeking. Workers who visited the GP had lower educational levels than non-visitors [6]. Lower social status and perceived inadequacy of income are independent risks for working people to seek consultation because of a new episode of LBP, especially for women [11]. Furthermore, women in the working population more often seek care than men [8,10]. Finally, risk patterns for seeking care because of neck or shoulder pain differ between the sexes [12-14] or were found only for men and not for women [10].

In addition to health and demographic factors, several work-related factors were found to be associated with medical care-seeking (studies on the whole working population except for 13). Low use of skills [15], poor job satisfaction [15] and not having a fixed salary [13] lead to more health care visits. These differ between the sexes. In men, poor job satisfaction, mostly routine work without opportunities for learning [12], and low demands in relation to competence [13] increased the risk for care-seeking. In women, reduced opportunities to acquire new knowledge [14], and solitary work [13] were risk indicators for seeking care. Another factor that can influence medical care-seeking is a worker’s working hours. For example, Josephson et al. [16] concluded that work itself is not a risk factor for care-seeking in women. They found no increased relative risk for care-seeking for low back pain and for neck/shoulder disorders for gainfully employed women compared to those who were not employed, or for women working full-time compared to those working part-time. However, at least 60 hours per week of paid work, or at least 40 hours per week of unpaid work, separately, indicated an increased relative risk for care-seeking. Furthermore, working at night was associated with GP consultation [8]. For men, occupational risk factors for seeking care because of neck or shoulder pain included night work/shift work, and working alone [10]. Although physical workload is expected to be a cause of back and/or neck and shoulder complaints, and thus a reason for seeking health care, studies on the relation between work characteristics and care-seeking show varying results. IJzelenberg and Burdorf [8], for example, found that well-known work-related risk factors for the occurrence of LBP did not determine the use of care for workers with LBP. In addition, heavy physical work was not an independent risk factor for care-seeking in workers because of neck or shoulder disorders [13] or LBP [9]. However, several other studies found a relation between physical workload and an increased use of health care. Delsasso-van de Kamp [17], for example, found that a demanding physical workload was the most important determinant for visiting a GP among military personnel. In addition, work that required strenuous use of the arms was associated with referral to a physical therapist [8], and a physical workload perceived to be high was found to be associated with LBP-related health care use [8,16]. Finally, risk factors related to posture and vibration were associated with more workers visiting the GP and medical specialist. [15].

Some studies found differential effects for men and women. Among women, an increased amount of visual display terminal (VDT) work, work above shoulder level [14], repetitive hand or finger movements and constrained sitting were indicators for seeking care [13]. Vingard et al. [12] found that high physical loads, in general, were related to increased relative risks for care-seeking for LBP in the general working population, but only for women. Among men, an increased amount of seated work [14], manual tasks, hindrances at work, working alone [10] or working with vibrating tools were found to be risk indicators for seeking care because of neck and shoulder disorders [13]. In addition, physical load from bending forward was related to increased relative risks for care-seeking for LBP in the general working population, but only for men [12].

Emotional or psychological work demands can play a role in relation to care-seeking. High levels of perceived job strain [8,15] or high levels of psychological job demands [6] were found to be associated with more care-seeking. However, Mortimer et al. [9] found that job strain did not affect care-seeking behaviour for LBP. With regard to decision latitude and control it was found that lower levels of decision latitude or a diminished sense of control at work can be associated with more visits to health care providers [6,7,15]. Finally, three studies [6,8,15] found that low levels of social support at work were related to more health care use.

### Purpose of the study

The findings of studies on care-seeking behaviour in relation to work characteristics, are sometimes inconclusive or even contradictory. It is not clear whether, or which, work characteristics are independent predictors of health care-seeking. This can be due to differences in study populations, measurements, and methods of analysing data. Therefore, in this study we will investigate the independent attribution of work characteristics to medical care- seeking in Dutch workers with health complaints at baseline, after controlling for personal, health and sickness absence related factors, in a longitudinal dataset.

## Methods

### Design

We performed a longitudinal two-wave study among a sample of Dutch workers, 15–64 years of age with a labour contract for 12 hours or more. Data were gathered through an existing internet panel run by a large market research organization. In order to obtain reliable estimates of care-seeking behaviour in relation to health, we required a sufficient number of participants with health complaints, chronic disease and/or a recent history of sickness absence. Therefore, participants were selected by a stratified procedure. In September 2005 the market research bureau sent a screener to 73,777 workers included in their panel. This resulted in 32,919 responses containing information about having a chronic disease (yes/no), health complaints (yes/no), the interference of health complaints with work (yes/no) and duration of sickness absence in the last six months (none, up to one week, longer than one week). Based on the screener these workers were assigned to one of 15 groups. They all received an e-mail invitation to participate in the study. A reminder was sent after one week and after two weeks. Each of the 15 groups was closed when the number of responses was high enough. In order to prevent certain workers from having a greater chance of responding to the invitation than others, half of the group was approached during weekdays and the other half during the weekend.

At the first measurement, 3048 participants filled out the internet questionnaire. Questions about care-seeking covered the period 1 June 2005 to 15 December 2005. These 3048 participants were approached again by e-mail in June 2006 (response 79.9%; questions about care-seeking covered the period 15 December 2005 to 1 June 2006). Thus, the total study period is one year.

Participants who reported conflicting demographic variables (e.g. gender, age) at different measurements, participants whose demographic variables differed from those in the files of the market research organization, and women who were pregnant during this study (since they almost always contact a physician) were deleted from the data file. For the analyses we selected the subjects who responded to two waves. This left us with a final dataset of 2305 subjects. Approval by a Medical Ethical Commission was not necessary under Dutch law.

### Sample

The sample for this study is largely representative of the population of Dutch workers, and participants from all major classes of occupations and branches of industry were included. We developed a weight factor to adjust for the sample selection procedure in comparison to the Dutch working population with respect to gender, age and educational level. After weighing, compared to the Dutch working population (Statistics Netherlands), the study population consisted of fewer workers in the age group 15–34 years (32% vs. 35%), fewer workers with a low educational level (20% vs. 24%) and fewer immigrants (8% vs. 17%).

Of the 2305 participants, 40% were female and 60% were male. The mean age at the first measurement was 40 years (SD =10.4). Educational level was low for 20% of the participants (no education beyond primary school), intermediate for 45% of the participants (intermediate secondary, higher secondary, intermediate vocational education or pre-university school), and high for 35% of the participants (higher vocational training or university). At the first measurement, most participants (78%) held a permanent job with their current employer. The others worked in temporary jobs, were temporary or freelance workers, or self-employed, Thirty-six per cent of the participants had a part-time contract of 35 hours a week or less.

### Questionnaire

The questionnaire was virtually identical in both measurements. It contained a large number of questions on several topics concerning personal characteristics (age, education, coping style), health (type of health complaint, health status, control over health outcomes, fatigue), sickness absence (rate and frequency), work characteristics (working hours, burnout, engagement, social support, physical, static and emotional workload, work pressure and decision latitude),, the relation between health complaints and work (work handicap), and use of health care (contact with a health care practitioner) (see Additional file: 1).

### Measures

#### Outcome measures

The outcome measures were defined as visiting a general practitioner (GP), a physical therapist, a medical specialist and/or visiting a mental health professional(second wave). Physical therapists include physiotherapists, Mensendieck therapists, Cesar therapists, manual therapists, podiatrists, and other physical therapists. Medical specialists include neurologists, orthopaedists, surgeons, rheumatologists, internists, plastic surgeons and other specialists. Mental health professionals include psychiatrists, psychologists, and social workers.

For all outcome measures we calculated a dichotomous measure (visit yes/no) and the logarithm of the average frequency of visits.

#### Personal characteristics

Demographic information was collected on gender, age and educational level. Educational level reflected the highest level of education completed and was divided into three categories: lower, intermediate and higher level. Age was divided into four categories: 15–30, 31–40, 41–50 and 51–64 years.

For questions about coping we used a shortened version of the Utrecht Coping List (UCL) [18]. Three *coping styles* were distinguished: avoidance behaviour (3 items), support-seeking (3 items) and active problem solving (3 items). All items have four response categories (Cronbach’s alpha 0.73, 0.74 and 0.71 respectively) and scale responses were dichotomized around the median (1.85, 2.45 and 3.15 respectively).

#### Health characteristics

The questionnaire included questions concerning general health, chronic disease, health problems, fatigue and burnout.

*Self-rated health status* was assessed by an overall rating of health on a five-point scale, with 1 = excellent and 5 = poor. The responses were dichotomized, with 1–3 indicating excellent/(very)good health and 4–5 indicating average/poor health.

Information on *chronic disease* was obtained by two questions: ‘Do you have a long-term disease, disorder or condition?’ with five listed categories (none, mental, musculoskeletal, other disease, co-morbidities); and if ‘yes’, ‘Does this disease, disorder or condition give you long-term health problems?’

Information on *current health problems* was obtained by two questions: ‘At this moment, do you have health problems’; and if ‘yes’, ‘Which of the following health problems do you have at this moment and since when (month, year)’ with 3 musculoskeletal, 3 mental health problems and 9 other health problems listed. In addition, subjects were asked whether their health problems (current or due to a chronic disease) interfered with their work performance *(health-related work problems*). Health complaints were divided into three categories: musculoskeletal, psychological and other. Having a chronic disease was defined as having health complaints before the onset of the study; other health complaints were defined as ‘new health complaints’. New health complaints are reported in the descriptives. Because of the low sample size and the relative long existence of these complaints (often more than three months), these subjects were merged with workers with chronic health complaints.

*Fatigue* was assessed using the Checklist Individual Strength (CIS), a self-report instrument consisting of four scales based on different modes of expressing fatigue (fatigue severity, reduced motivation, reduced activity, reduced concentration) [19,20]. The reference period for the scale was the past two weeks. Total sum scores ranged from 20 to 80. A higher score implies more fatigue (Cronbach’s alpha = 0.94).

A 3-item subscale of the Multidimensional Health Locus of Control Scales [21] was used to assess a person’s beliefs about control over health outcomes (‘*Locus control own health’*)*.* A higher score indicates more perceived control over one’s own health (Cronbach’s alpha = 0.68).

### Sickness absence

In each questionnaire participants were asked to report the number of sickness absence spells during the past six months, the starting and end date of each spell, including embedded non-working days and weekend days. We used sick rate and the number of sickness absence spells.

### Work characteristics

*Engagement* is a concept that refers to being fully immersed in an activity (absorption), being highly activated (vigour) and to identify with the work (dedication), and is an indicator of both a high level of motivation for work and high levels of energy. We used only the four items in the dedication subscale of the engagement scale of Schaufeli et al. [22]. The items have five response categories, ranging from (1) never to (5) always. A higher score indicates more work dedication (Cronbach’s alpha = 0.90).

*Burnout* was measured with the five-item emotional exhaustion scale of the Dutch version of the Maslach Burnout Inventory [23]. Answer categories varied from (1) never to (5) almost daily. A higher score indicates more exhaustion (Cronbach’s alpha = 0.89).

Psychosocial work factors were measured by the Dutch version of the Job Content Questionnaire [24], which measures *work pressure* (4 items), *decision latitude* (4 items), *social support from co-workers* and *social support from supervisor* (4 items each), Cronbach’s alpha = 0.80, 0.84, 0.81 and 0.88).

*Emotional workload* was measured by the sum of 3 items. The questions originated from the Dutch version of the Copenhagen Psychosocial Questionnaire and responses varied from (1) never to (4) always [25] (Cronbach’s alpha = 0.80).

Physical work demands were measured by 5 items and divided into 2 subscales: *heavy physical workload* (carrying or lifting heavy weights of more than 5 kg and exerting force with arms or legs, work requiring repetitive movements over a long period of time) and *static physical workload* (work requiring holding the same posture over a long period of time and/or working with visual display units) (Cronbach’s alpha = 0.73 and 0.71).

A work handicap was defined as having health complaints that impeded work performance.

### Data analysis

Analyses were conducted using SPSS software, version 14.0 for Windows. The outcome measures were constructed using information from the second survey. The independent variables were constructed using the initial survey. In this way we circumvented the possible problem that the independent variables might explain not only the outcome measures, but also the other way around. This would lead to biased estimates [26] pp. 49–51]. Scales were dichotomized around the median value unless otherwise described.

Because different mechanisms are expected with regard to gender, we will present separate analyses for men and women in relation to care-seeking measures. Descriptives of men and women were compared using the Chi square test or *T*-test.

The effect of the independent variables on the dichotomous outcome measures was estimated using logistic regression models. In estimating the effect of the independent variables on the logarithm of the average yearly frequency of visits we used only information from subjects who visited a health care practitioner at least once. To test whether visitors differ fundamentally from non-visitors we performed the Heckman’s sample selection test [26] pp. 564–566]. The Heckman tests for the models for the logarithm of the average yearly frequency of visits in this research were all rejected. This means that there is no sample selection and that we are allowed to estimate ordinary least squares models for the logarithm of the average yearly frequency of visits. Only the yearly frequency of visits to the GP could be analysed. The sample size of other health care providers was too small to run regression analysis on frequencies. We planned to distinguish between new and chronic health complaints in the analyses. However, because of small sample sizes and no added value we omitted this variable from the analyses. All significant tests in this research are two-tailed.

## Results

### Sex differences in independent and outcome variables

The 2305 subjects consisted of 1393 men and 912 women.

Descriptive results and significant differences between men and women for independent variables are shown in Table 1. Men in this sample were slightly older, more highly educated, and more often had an active, less avoiding and less support-seeking coping style than women. When considering health characteristics, we found that a higher percentage of women than men reported musculoskeletal and other health complaints, a lower health status, a lower perceived locus of control over their health, and higher levels of fatigue. Finally, work characteristics were also found to differ between men and women. A higher percentage of women worked in part-time jobs, reported a work handicap, had lower levels of engagement, decision latitude, and had both higher levels of social support from colleagues and greater emotional workloads than men. Women reported a higher number of absence spells (6.1 versus 5.2 times per year) and a higher absence rate (5.3 versus 3.5%) than men.

**Table 1:** Descriptives of independent variables

**Independent variable**	**% men (n = 1393)**	**% women (n = 912)**	**Sig. Diff.**	**% total (n = 2305)**
**Personal characteristics**				
*Age * 15*–*30 years	*20.0*	*21.9*		*19.4*
31-40 years	29*.*7	33*.*8	*	31*.*9
41-50 years	31*.*4	27*.*2	*	29*.*7
51-64 years	18*.*9	17*.*1		19*.*1
*Education* Low	19*.*2	20*.*8		19*.*7
Intermediate	43*.*7	48*.*4	*	45*.*6
High	37*.*1	30*.*8	**	34*.*8
*Coping styles* More avoiding	47*.*8	54*.*5	**	49*.*7
More support seeking	30*.*1	53*.*3	***	39*.*3
More active	46*.*9	39*.*9	***	44*.*8
**Health**				
*New health complaints*				
Musculoskeletal	3*.*1	3*.*1		2*.*7
Psychological	0*.*9	2*.*1		1*.*3
Other	6*.*4	8*.*1		7*.*2
*Chronic health complaints*				
Musculoskeletal	20*.*7	31*.*4	***	24*.*9
Psychological	8*.*8	11*.*0		9*.*7
Other	24*.*3	30*.*8	***	26*.*9
Good health status	84*.*9	78*.*9	***	83*.*7
Good control over health outcomes	50*.*9	42*.*8	**	48*.*0
High fatigue	10*.*7	14*.*8	**	12*.*3
**Work characteristics**				
Full time work (40 hrs/wk or more)	43*.*8	15*.*1	***	32*.*4
High burnout	41*.*0	42*.*2		41*.*2
Work handicap	17*.*4	23*.*0	***	18*.*8
High engagement	58*.*9	54*.*7	*	57*.*8
High social support supervisor	44*.*6	45*.*4		45*.*6
High social support colleagues	47*.*1	53*.*2	**	50*.*1
High physical work load	53*.*9	52*.*9		53*.*4
High static work load	45*.*5	47*.*0		46*.*0
High work pressure	50*.*1	45*.*9		48*.*3
High emotional workload	46*.*7	54*.*1	***	49*.*0
High decision latitude	62*.*1	55*.*5	**	60*.*9

Sig = significant difference between men and women; * = p < 0.05; ** = p < 0.01; *** = p < 0.001.

Descriptive results for outcome variables are summarized in Table 2. Numbers in this table were calculated from the total sample, i.e. both waves, and cover the time span of one year. The GP was contacted by 68% of the workers during the follow-up period; 24% had contacted a physical therapist; 29% had contacted a medical specialist, and 10% had contacted a mental health professional.

**Table 2:** Descriptives of yearly care seeking for all workers

		**Men**			**Women**				**Total**				
**Care seeking**	**%**	**mean**	**sd**	**N**	**%**	**mean**	**sd**	**N**	**%**	**mean**	**sd**	**N**	**sig**
**General practitioner**													
- % with contact	61.7			1393	76.7			912	67.7			2305	***
- average yearly frequency^1^		3.0	3.8	860		3.4	4.2	700		3.2	4	1561	*
**Physical therapist**													
- % with contact	20.7			1393	28			912	23.6			2305	***
- average yearly frequency^1^		14.0	19.8	288		18.3	20.3	256		16	20.2	544	*
**Medical specialist**													
-% with contact	24.6			1393	35.3			912	28.9			2305	***
- average yearly frequency^1^		4.2	8.4	343		3.7	4.7	322		4	6.9	665	
**Mental health professional**													
- % with contact	8.0			1393	12.6			912	9.8			2305	***
- average yearly frequency^1^		7.9	13.2	112		10.1	13.9	115		9	13.6	226	

^1^ = Frequencies were calculated for workers with contact only. This is why the number of observations is lower (N). Sig = significant difference between men and women; * = p < 0.05; *** = p < 0.001.

A higher percentage of women than men had been in contact with a GP, a medical specialist, a physical therapist or a mental health professional. The frequency of contacts with a health care provider only differed for the GP and physical therapist: when workers visited their GP or a physical therapist, women came back more frequently than men.

### Determinants of visits to a GP

The outcomes of the multivariate regression analyses are summarized in Tables 2009;3 (visits yes/no) and 4 (visit frequency). The personal characteristics that were related to contact with a health care provider were age and coping style for men. When age increased, more men visited the GP. Of men with a more avoiding coping style a lower percentage had contact with a GP. When workers had contact with a GP, the number of visits per year was related to age, and coping style. Older workers visited the GP more often than younger ones. In addition, women with a more active coping style and men with a more support seeking coping style made more frequent visits.

**Table 3:** Determinants of visits to a GP (yes/no) for men and women with health complaints

	**GP visits (yes/no)**					
	**Men (n = 619)**			**Women (n = 492)**		
**Variable (reference category)**	**OR**		**Confidence interval**	**OR**		**Confidence interval**
**Personal characteristics**						
Age (31–40 years)						
15-30 years	1.49		(0.88 - 2.53)	0.65		(0.37 - 1.13)
41-50 years	1.49	#	(0.96 - 2.31)	1.17		(0.72 - 1.91)
51-64 years	1.75	*	(1.09 - 2.82)	1.52		(0.85 - 2.72)
Education (Intermediate)						
Low	0.94		(0.59 - 1.49)	1.37		(0.82 - 2.31)
High	0.92		(0.62 - 1.35)	1.14		(0.72 - 1.81)
Coping styles Avoiding (low)	0.71	*	(0.50 - 1.00)	1.11		(0.74 - 1.67)
Active (low)	0.98		(0.68 - 1.42)	1.09		(0.74 - 1.61)
Support seeking (low)	1.02		(0.72 - 1.45)	1.06		(0.71 - 1.58)
**Health**						
Health complaints (other)						
Musculo skeletal	0.81		(0.58 - 1.14)	1.31		(0.88 - 1.95)
Psychological	1.01		(0.63 - 1.61)	1.50		(0.90 - 2.52)
Health status (low)	0.68	#	(0.45 - 1.03)	0.88		(0.55 - 1.40)
Control over health outcomes (low)	0.81		(0.58 - 1.15)	1.04		(0.69 - 1.56)
Fatigue (low)	1.65	#	(0.98 - 2.76)	1.02		(0.60 - 1.72)
**Sickness absence**						
Sickness absence (%)	0.99		(0.98 - 1.00)	0.99	*	(0.98 - 1.00)
Sickness absence (frequency)	1.09		(0.90 - 1.33)	0.98		(0.79 - 1.22)
**Work characteristics**						
Full time work (<40 hrs/wk)	1.01		(0.72 - 1.43)	1.53		(0.88 - 2.69)
Burnout (low)	0.91		(0.62 - 1.35)	0.71		(0.45 - 1.10)
Work handicap (no)	0.99		(0.67 - 1.44)	1.60	*	(1.03 - 2.49)
Engagement (low)	1.49	*	(1.02 - 2.19)	0.83		(0.55 - 1.27)
Social support supervisor (low)	0.91		(0.63 - 1.32)	1.19		(0.79 - 1.79)
Social support colleagues (low)	0.91		(0.64 - 1.29)	0.96		(0.64 - 1.43)
Work load physical (low)	1.21		(0.84 - 1.75)	0.92		(0.62 - 1.38)
Work load static (low)	1.01		(0.71 - 1.43)	1.02		(0.68 - 1.53)
Work pressure (low)	1.08		(0.76 - 1.54)	0.96		(0.64 - 1.44)
Emotional workload (low)	0.99		(0.69 - 1.41)	0.86		(0.57 - 1.29)
Decision latitude (low)	0.96		(0.66 - 1.38)	0.69	#	(0.46 - 1.05)
R Square Personal characteristics	2.2			2.1		
R Square Health characteristics	2.0			1.5		
R Square sickness absence	0.4			1.0		
R Square Work characteristics	1.3			3.4		
R Square Total	5.9	#		8.0	***	

# = p < 0.10; * = p < 0.05; ** = p < 0.01; *** = p < 0.001.

^1^Nagelkerke R Square.

**Table 4:** Determinants of visits to a GP (frequency) for men and women with health complaints

	**GP visits (frequency/year)**			
	**Men (n = 330)**		**Women (n = 299)**	
**Variable (reference category)**	**beta**		**beta**	
**Personal characteristics**				
Age (31–40 years)				
15-30 years	−0.077		0.018	
41-50 years	0.049		0.105	
51-64 years	0.198	**	0.164	*
Education (Intermediate)				
Low	0.025		−0.040	
High	0.024		−0.118	#
Coping styles Avoiding (low)	0.062		−0.072	
Active (low)	0.022		0.135	*
Support seeking (low)	0.136	*	0.095	
**Health**				
Health complaints (other)				
Musculo skeletal	0.021		0.007	
Psychological	0.096		−0.013	
Health status (low)	−0.050		−0.126	#
Control over health outcomes (low)	0.041		−0.071	
Fatigue (low)	0.008		0.197	**
**Sickness absence**				
Sickness absence (%)	−0.055		0.039	
Sickness absence (frequency)	0.099	#	0.051	
**Work characteristics**				
Full time work (<40 hrs/wk)	0.147	**	−0.013	
Burnout (low)	0.067		−0.085	
Work handicap (no)	0.005		0.018	
Engagement (low)	0.016		−0.061	
Social support supervisor (low)	−0.079		0.031	
Social support colleagues (low)	−0.150	**	0.000	
Work load physical (low)	0.002		−0.040	
Work load static (low)	−0.083		0.009	
Work pressure (low)	−0.095	#	−0.021	
Emotional workload (low)	−0.007		0.023	
Decision latitude (low)	−0.216	***	−0.069	
R Square Personal characteristics	3.6	*	2.6	*
R Square Health characteristics	0.9		7.1	**
R Square sickness absence	0.5		0.0	
R Square Work characteristics	8.0	*	−1.9	
R Square Total	13.0	***	7.8	**

# = p < 0.10; * = p < 0.05; ** = p < 0.01; *** = p < 0.001.

^1^Adjusted R Square.

Health care characteristics only slightly contributed to contact with a GP, once again differently for men and women. Relatively more men visited a GP when they reported a a low health status or high levels of fatigue. If women had contact with a GP the frequency of these contacts was higher when they experienced a lower health status or high levels of fatigue.

Sickness absence rate was related to less contact with the GP but only for women. Sickness absence frequency was related to a higher contact frequency but only for men.

Given personal characteristics, health and sickness absence, work characteristics were related to contact with a GP. High levels of engagement were related to more men visiting the GP and experiencing a work handicap or low levels of decision latitude were related to more women visiting the GP. When workers had contact with a GP, men went more frequently when they worked fulltime, and when they reported low social support from colleagues, low levels of work pressure and low levels of decision latitude. For women, work charateristics were not found to be related to the frequency of visits to the GP.

If we focus especially on the partial contribution of work characteristics, we find that work characteristics significantly and independently contributed to the frequency of visiting a GP, but only for men.

### Determinants of visits to a physical therapist

Although both models are significant, determinants of visiting a physical therapist mostly show weak relations (p < 0.10, Table 5). A higher percentage of men visited the physical therapist when they experienced high levels of fatigue, a higher sickness absence rate, a work handicap, high levels of emotional workload and decision latitude. A higher percentage of women visited the physical therapist when they were over 50 years of age report higher sickness absence rates, experienced low levels of fatigue or low levels of burnout. Most important result is that when men experienced a work handicap, a higher percentage visited the physical therapist.

**Table 5:** Determinants of visits to a physical therapist (yes/no) for men and women with musculoskeletal health complaints

	**Visits to a physical therapist (yes/no)**					
	**Men (n = 325)**			**Women (n = 311)**		
**Variable (reference category)**	**OR**		**Confidence interval**	**OR**		**Confidence interval**
**Personal characteristics**						
Age (31–40 years)						
15-30 years	0.50		(0.20 - 1.26)	0.85		(0.38 - 1.92)
41-50 years	0.76		(0.38 - 1.50)	1.53		(0.79 - 2.96)
51-64 years	0.62		(0.29 - 1.32)	1.88	#	(0.93 - 3.78)
Education (Intermediate)						
Low	1.03		(0.52 - 2.05)	0.63		(0.32 - 1.23)
High	0..82		(0.43 - 1.56)	0.71		(0.37 - 1.36)
Coping styles Avoiding (low)	0.78		(0.45 - 1.37)	0.96		(0.56 - 1.66)
Active (low)	0.74		(0.39 - 1.40)	1.03		(0.62 - 1.71)
Support seeking (low)	0.95		(0.55 - 1.63)	1.13		(0.66 - 1.94)
**Health**						
Health status (low)	1.43		(0.73 - 2.77)	0.63		(0.35 - 1.15)
Control over health outcomes (low)	1.37		(0.79 - 2.40)	1.03		(0.60 - 1.75)
Fatigue (low)	2.02	#	(0.95 - 4.27)	0.51	#	(0.25 - 1.04)
**Sickness absence**						
Sickness absence (%)	1.01	#	(1.00 - 1.03)	1.01	#	(1.00 - 1.02)
Sickness absence (frequency)	0.80		(0.58 - 1.11)	0.87		(0.65 - 1.16)
**Work characteristics**						
Full time work (<40 hrs/wk)	0.72		(0.42 - 1.23)	1.11		(0.52 - 2.34)
Burnout (low)	0.67		(0.36 - 1.23)	0.59	#	(0.33 - 1.06)
Work handicap (no)	2.43	**	(1.29 - 4.60)	1.33		(0.75 - 2.37)
Engagement (low)	1.16		(0.63 - 2.13)	0.73		(0.42 - 1.27)
Social support supervisor (low)	1.20		(0.67 - 2.16)	1.05		(0.62 - 1.78)
Social support colleagues (low)	0.87		(0.50 - 1.52)	1.11		(0.66 - 1.88)
Work load physical (low)	1.00		(0.56 - 1.77)	0.77		(0.45 - 1.31)
Work load static (low)	0.90		(0.52 - 1.56)	0.82		(0.49 - 1.38)
Work pressure (low)	1.18		(0.66 - 2.11)	1.17		(0.69 - 1.98)
Emotional workload (low)	1.74	#	(0.98 - 3.07)	1.05		(0.61 - 1.82)
Decision latitude (low)	1.70	#	(0.94 - 3.06)	0.84		(0.49 - 1.44)
R Square Personal characteristics	2.5			3.3		
R Square Health characteristics	1.4			2.0		
R Square sickness absence	2.1	#		2.0		
R Square Work characteristics	7.1	#		2.6		
R Square Total	13.1	***		9.9	***	

# = p < 0.10; * = p < 0.05; ** = p < 0.01; *** = p < 0.001.

^1^Nagelkerke R Square.

### Determinants of visits to a medical specialist

Table 6 gives the results for visiting a medical specialist. For men, we found that personal characteristics were most important whereas for women it was health that was most related to visiting a medical specialist. A higher percentage of men visited the medical specialist when they were younger or older than 31–40 years of age, when they reported low levels of avoidance and/or when they did not report psychological health complaints. One work characteristic was related to a lower percentage of men visiting a medical specialist: high levels of support from the supervisor. Less women visited the medical specialist when they experienced a high health status, high levels of burnout and/or engagement.

**Table 6:** Determinants of visits to a medical specialist (yes/no) for men and women with health complaints

	**Visits to a medical specialist (yes/no)**					
	**Men (n = 619)**			**Women (n = 492)**		
**Variable (reference category)**	**OR**		**Confidence interval**	**OR**		**Confidence interval**
**Personal characteristics**						
Age (31–40 years)						
15-30 years	2.05	*	(1.02 - 4.13)	0.68		(0.35 - 1.31)
41-50 years	2.37	**	(1.33 - 4.21)	1.23		(0.72 - 2.11)
51-64 years	2.60	**	(1.41 - 4.81)	1.11		(0.60 - 2.05)
Education (Intermediate)						
Low	1.13		(0.65 - 1.95)	0.86		(0.49 - 1.52)
High	1.27		(0.79 - 2.04)	1.21		(0.72 - 2.02)
Coping styles Avoiding (low)	0.56	**	(0.37 - 0.86)	0.74		(0.47 - 1.16)
Active (low)	1.00		(0.64 - 1.55)	0.86		(0.56 - 1.32)
Support seeking (low)	1.17		(0.77 - 1.78)	0.98		(0.63 - 1.53)
**Health**						
Health complaints (other)						
Musculo skeletal	1.16		(0.75 - 1.80)	1.21		(0.78 - 1.89)
Psychological	0.34	**	(0.16 - 0.71)	0.65		(0.34 - 1.26)
Health status (low)	0.82		(0.50 - 1.32)	0.48	**	(0.29 - 0.79)
Control over health outcomes (low)	0.97		(0.64 - 1.47)	0.98		(0.63 - 1.54)
Fatigue (low)	1.47		(0.78 - 2.76)	1.12		(0.64 - 1.98)
**Sickness absence**						
Sickness absence (%)	1.01		(0.99 - 1.02)	0.99		(0.98 - 1.01)
Sickness absence (frequency)	1.14		(0.91 - 1.44)	1.14		(0.90 - 1.44)
Work characteristics						
Full time work (<40 hrs/wk)	0.93		(0.61 - 1.42)	1.07		(0.58 - 1.99)
Burnout (low)	0.86		(0.54 - 1.35)	0.64	#	(0.39 - 1.05)
Work handicap (no)	1.44		(0.91 - 2.27)	0.96		(0.59 - 1.56)
Engagement (low)	1.37		(0.86 - 2.18)	0.64	#	(0.40 - 1.02)
Social support supervisor (low)	0.63	*	(0.40 - 0.98)	1.31		(0.84 - 2.06)
Social support colleagues (low)	1.16		(0.76 - 1.78)	0.98		(0.63 - 1.53)
Work load physical (low)	0.88		(0.57 - 1.37)	1.09		(0.70 - 1.70)
Work load static (low)	0.77		(0.51 - 1.18)	0.81		(0.52 - 1.27)
Work pressure (low)	0.77		(0.51 - 1.18)	0.99		(0.64 - 1.54)
Emotional workload (low)	0.89		(0.57 - 1.37)	0.83		(0.52 - 1.30)
Decision latitude (low)	1.10		(0.70 - 1.72)	0.79		(0.51 - 1.24)
R Square Personal characteristics	6.80	***		1.6		
R Square Health characteristics	2.40	#		3.1	#	
R Square sickness absence	0.80			0.5		
R Square Work characteristics	2.90			3.1		
R Square Total	12.90	***		8.3	***	

# = p < 0.10; * = p < 0.05; ** = p < 0.01; *** = p < 0.001.

^1^Nagelkerke R Square.

### Determinants of visits to a mental health professional

Although the total R square is high, individual variables show few significant results (Table 7). This may be due to the small sample size. For men, health characteristics were independently related to visiting a mental health professional. More women visited a mental health professional when they had a low educational level and when they experienced low levels of burnout and/or a work handicap.

**Table 7:** Determinants of visits to a mental health professional (yes/no) for men and women with psychological health complaints

	**Visits to a mental health professional (yes/no)**					
	**Men (n = 133)**			**Women (n = 114)**		
**Variable (reference category)**	**OR**		**Confidence interval**	**OR**		**Confidence interval**
**Personal characteristics**						
Age (31–40 years)						
15-30 years	0.53		(0.09 - 3.32)	0.30		(0.05 - 1.77)
41-50 years	1.14		(0.27 - 4.79)	1.09		(0.35 - 3.42)
51-64 years	0.73		(0.15 - 3.46)	0.64		(0.12 - 3.36)
Education (Intermediate)						
Low	0.56		(0.09 - 3.55)	3.76	*	(1.03 - 13.75)
High	1.18		(0.34 - 4.15)	0.94		(0.28 - 3.18)
Coping styles Avoiding (low0	1.65		(0.53 - 5.14)	0.87		(0.30 - 2.52)
Active (low)	1.87		(0.59 - 5.92)	0.87		(0.32 - 2.35)
Support seeking (low)	0.50		(0.16 - 1.62)	2.02		(0.69 - 5.87)
**Health**						
Health status (low)	1.59		(0.52 - 4.88)	0.68		(0.22 - 2.11)
Control over health outcomes (low)	2.13		(0.64 - 7.08)	0.71		(0.25 - 2.00)
Fatigue (low)	2.55		(0.63 - 10.31)	1.40		(0.45 - 4.37)
**Sickness absence**						
Sickness absence (%)	1.00		(0.98 - 1.02)	1.01		(1.00 - 1.03)
Sickness absence (frequency)	0.97		(0.55 - 1.70)	1.02		(0.63 - 1.66)
**Work characteristics**						
Full time work (<40 hrs/wk)	0.50		(0.15 - 1.63)	1.46		(0.42 - 5.06)
Burnout (low)	2.77		(0.44 - 1.39)	0.34	#	(0.10 - 1.13)
Work handicap (no)	1.72		(0.48 - 6.21)	3.08	#	(0.90 - 10.51)
Engagement (low)	0.77		(0.18 - 3.31)	1.95		(0.65 - 5.82)
Social support supervisor (low)	1.49		(0.39 - 5.63)	2.37		(0.70 - 8.00)
Social support colleagues (low)	0.87		(0.25 - 2.98)	0.50		(0.17 - 1.46)
Work load physical (low)	0.45		(0.14 - 1.40)	0.58		(0.21 - 1.59)
Work load static (low)	1.38		(0.47 - 4.10)	2.31		(0.83 - 6.38)
Work pressure (low)	0.78		(0.22 - 2.79)	1.12		(0.39 - 3.18)
Emotional workload (low)	2.02		(0.51 - 7.94)	1.50		(0.45 - 4.98)
Decision latitude (low)	2.51		(0.82 - 7.63)	1.14		(0.41 - 3.22)
R Square Personal characteristics	6.5			9.5		
R Square Health characteristics	9.3	*		2.3		
R Square sickness absence	0.3			3.9		
R Square Work characteristics	10.6			16.3		
R Square Total	26.7	***		32.0	**	

# = p < 0.10; * = p < 0.05; ** = p < 0.01; *** = p < 0.001.

^1^Nagelkerke R Square.

## Discussion

This study shows that in the Dutch working population, when workers report - often long-term - health complaints, personal, health, and work characteristics, but not sickness absence, are associated with later care-seeking.

We expected to find gender differences in care-seeking. We found that women experience more chronic health complaints and seek health care more often than men, which is in agreement with many other studies (see Introduction). Once a health care provider was contacted the frequency of visits did not differ between the sexes for the medical specialist and mental health professional, but it did for the GP and the physical therapist. Women visited these more frequently than men.

The influence of coping styles on care-seeking differed interestingly between men and women. Men with a predominantly avoiding coping style sought care less often from a GP or medcial specialist, whereas men with a support seeking coping style visited the GP more often. Women with an active coping style visited the GP more frequently. This result is in agreement with Mortimer et al. [9] as far as women are concerned: she found that the use of passive coping strategies increased the probability of *not* seeking care, but only for women, not for men. Our results suggest that women actively seek care, whereas men either avoid medical help altogether or seek support from the GP. Corney [27] found that psychosocial problems or distress predicted consultation behaviour in women, but not in men, and suggested that women found it easier to divulge personal information to others than men did. This may be an explanation why in our study men with mental health problem seek less care from a medical specialist. An important obstacle to improving men's health is their apparent reluctance to consult a doctor. Men with health problems are more likely than women to have had no recent contact with a doctor regardless of income or ethnicity. This reluctance means that men often do not seek help until a disease has progressed, which can have serious consequences. Gouwy et al. [28] concludes that men do care about health issues but often find it difficult to expresses their fears. Men also tend to attend their general practitioner later in the course of a condition than women.

Sickness absence was not an independent predictor of later care-seeking. It is expected that workers who are absent from work experience health complaints and seek medical care which explains the association between high medical consumption and high rates of sick rate [1-5]. Our study indicates that, after correction for personal and health characteristics, sickness absence does not predict later care-seeking for workers who already experience health complaints.

Next, we investigated whether work characteristics independently attributed to medical care-seeking. Although the contribution of work characteristics to care-seeking was sometimes higher than that of personal- or health related characteristics, work characteristics did only independently contribute to later care- seeking for the frequency of visiting the GP by men. We will discuss the association between individual work characteristics and care-seeking in the next paragraphs. We expected that working many hours per week would increase medical care-seeking. However, the number of working hours did not influence the decision to visit a care-practitioner but it increased the frequency of visits to a GP for men. This suggests a healthy worker effect: workers without health problems are simply able to work more hours. Apart from health, men and women have different reasons for working part-time. For example, in the Dutch working population women who are single parents work full-time more often than women with a partner and children at home (24% vs. 9%) whereas the opposite effect can be seen among men (63% vs. 89%).

Low levels of social support at work were expected to be related to more care-seeking, as was found in other studies [6,8,15,29]. We found only two effects for men. A high level of social support by colleagues was associated with a lower frequency of visiting a GP and a high level of social support by the supervisor was associated with a lower percentage of men who visited the medical specialist.

Results on burnout, workload, work pressure and decision latitude were not as expected. High levels of burnout were expected to increase seeking care, but we found no such results. The effect of burnout may be largely covered by fatigue (included in health characteristics) which was associated with later care-seeking with the GP and physical therapist. We did find that high levels of dedication to work were associated with more men visiting the GP. High levels of decision latitude for men led to a lower visiting frequency to the GP. Other studies found inconclusive relationships between physical workloads and care-seeking. We have to realize that the effects that we found exist after correcting for personal and health characteristics and sickness absence. Thus, the effect of physical workloads may be largely covered by health complaints and fatigue, whereas the remaining effect may be due to the perception of the workload. Furthermore, the nature of employment tends to differ for men and women, with men being more often involved in demanding physical work. The effects of physical workload should be studied within sectors to gain more insight into the relation between physical workload and care-seeking.

The only other studies restricted to workers with health complaints showed that high disability and high pain [9] or the number of symptoms [10] were most important when predicting care-seeking. Im this study we did not measure high pain or the severity of health complaints. However, we assume that the combination of health characteristics that we measured included an indication of severity. High disability may be compatible with our measurement of experiencing a work handicap. Experiencing a work handicap (health complaints that impede work performance) was significantly associated with more women visiting the GP and more men visiting a physical therapist and weakly associated with more women visiting a mental health professional. The predictive value of work characteristics on medical care-seeking differs between health care providers. Of all work characteristics, experiencing a work handicap and the perception of social support seem to be important factors. If we aim at reducing health care costs for workers, it is important to reduce the number of workers that report that health complaints impede their work performance. Steenbeek et al. [30] showed that employees with a work handicap differ considerably from employees with chronic health complaints. Employees with a work handicap have the worst working conditions and health, and they drift into even less favourable working conditions and outcomes. Furthermore, they are vulnerable to other working conditions than healthy employees. Steenbeek et al. conclude that the supervisor should provide more social support, closely monitor workload in combination with work pressure and decision latitude, and when possible help to adjust working conditions. This is in line with Nieuwenhuijsen et al. [31] who found that better communication between supervisor and employees absent due to mental health problems, was associated with favourable full return to work rates in non-depressed employees. Health care providers can reduce medical costs by taking the work relatedness of health complaints into account [32,33] and act accordingly. Costs of non-participation can be further reduced by decreasing the time to referral [33-35], by decreasing waiting lists [33-35] and by providing appropriate care and avoiding unnecessary or harmful care [36].

A strength of this study is the longitudinal data collection in a largely representative sample of Dutch workers. Many variables of interest were measured such as new and chronic health complaints, coping, work characteristics, all in combination with all dates of visits to health care practitioners.

Nevertheless, the study also suffers from some limitations. Regarding our sample selection method, three types of selection bias may have influenced our statistical results. First, data were collected by a self-reported questionnaire. This method always yields the danger of recall bias. Second, because we used an internet panel as the base-population, our study may suffer from a selective inclusion of participants familiar with the use of PCs and the Internet. Dutch national statistics at the time show that women and persons with lower educational background use the Internet significantly less than men and persons with a higher educational background. Third, an additional selection occurred by excluding data of several participants in the analysis.

## Conclusions

We can conclude that personal and health characteristics are most important when explaining medical care-seeking in the Dutch working population. Work characteristics independently attributed tomedical care-seeking but only for men and only for the frequency of visits to the GP. The association between work characteristics and later medical care-seeking differed between health care providers and between men and women. If we aim at reducing health care costs for workers by preventing unnecessary or inefficient care, it is important to reduce the number of workers that report that health complaints impede their work performance. The supervisor should provide more social support, closely monitor workload in combination with work pressure and decision latitude, and when possible help to adjust working conditions. Health care providers can reduce medical costs by taking the work relatedness of health complaints into account and act accordingly, by decreasing the time to referral and waiting lists, and by providing appropriate care and avoiding unnecessary or harmful care.

## Competing interests

The author declares that she has no competing interests.

## Authors' contributions

RS carried out the whole study.

## Pre-publication history

The pre-publication history for this paper can be accessed here:

http://www.biomedcentral.com/1472-6963/12/294/prepub

## Supplementary Material

Additional file 1 English translation of the original Dutch questionnaire for workers. Click here for file
