# Supplementary material for: The importance of job characteristics in determining medical care-seeking in the Dutch working population, a longitudinal survey study
Source: BMC Health Serv Res. 2012 Aug 31;12:294. doi: 10.1186/1472-6963-12-294 (PMC3444386; doi:10.1186/1472-6963-12-294)
Supplement: Additional file 1 — English translation of the original Dutch questionnaire for workers. [file 1472-6963-12-294-S1.doc]

| **QUESTIONNAIRE**  **Worker’s opinion on health, health care providers, care-seeking and sickness absence**  2006-2007 (translated from Dutch) |
| --- |

The purpose of this study is to acquire knowledge about workers’ care-seeking. When workers have health complaints, do they visit a health care provider and if they do who do they choose? Health-care providers are for example the general practitioner (GP), medical specialist, occupational physician (OP), mental health therapist and physiotherapist. We also like to know the worker’s opinion about the quality of care. We will ask how often a worker contacted which health-care provider and when, if these contacts contributed to improved health, less sickness absence and/or to return to work.

Respondents filled in an electronic version of the questionnaire. Depending on their answers they received only relevant questions. For example, if a respondent reported contact with the GP and OP, only questions addressing these visits were asked. This translated questionnaire is a summary. A category of possible questions is placed between brackets. For example [health care provider] means that the questions were asked for all health care providers a certain respondent had visited. The routing is not visible in this version. For example, questions about a supervisor were only presented to respondents who had a supervisor.

| General questions | |
| --- | --- |
| Sex | ❑ Male  ❑ Female |
| Year of birth | …… |
| What is your household-composition? | ❑ Married or living together without children living at home ❑ Married or living together with children living at home  ❑ One-parent household  ❑ Single  ❑ Other |
| What is the monthly income of your household (euros)? | ❑ <1,000  ❑ 1,000 – 1,999  ❑ 2,000 – 2,999  ❑ 3,000 – 3,999  ❑ 4,000 – 4,999  ❑ 5,000 or more |
| What is your highest educational level? | ❑ No education beyond primary school  ❑ Intermediate secondary, higher secondary, intermediate vocational education or pre-university school  ❑ Higher vocational training or university- |
| What is your type of employment? | ❑ Permanent position  ❑ Temporary position  ❑ Agency staff  ❑ Self-employed |
| Do you have a supervisor or work with colleagues (possibly as a supervisor)? | ❑ I have no supervisor nor colleagues  ❑ I have colleagues but no supervisor  ❑ I have a supervisor but no colleagues  ❑ I have both a supervisor and colleagues |
| What is your professional group? | ❑ Agriculture, fisheries, food industry  ❑ Construction and timber  ❑ Industry  ❑ Retail and wholesale  ❑ Transport  ❑ (Financial) services  ❑ Temp agencies  ❑ Healthcare  ❑ Government (education)  ❑ Government (other)  ❑ Other trades and professions |

| Coping | | | | |
| --- | --- | --- | --- | --- |
| Please indicate how you normally react to problems or unpleasant events | Almost never | Sometimes | Often | Very often |
| Yield to avoid difficult situations | ❑ | ❑ | ❑ | ❑ |
| Look at a problem from different angles | ❑ | ❑ | ❑ | ❑ |
| Show your feelings | ❑ | ❑ | ❑ | ❑ |
| Avoid difficult situations | ❑ | ❑ | ❑ | ❑ |
| Think of different possibilities to solve a problem | ❑ | ❑ | ❑ | ❑ |
| Seek consolation and understanding | ❑ | ❑ | ❑ | ❑ |
| Try to withdraw from the situation | ❑ | ❑ | ❑ | ❑ |
| Work purposely to solve a problem | ❑ | ❑ | ❑ | ❑ |
| Show your concerns | ❑ | ❑ | ❑ | ❑ |

| Dedication | | | | | |
| --- | --- | --- | --- | --- | --- |
| Please indicate how often each of the following statements applies to you: | Never | Rarely | Sometimes | Often | Always |
| I regard the work I do as useful and meaningful. | ❑ | ❑ | ❑ | ❑ | ❑ |
| I am enthusiastic about my job. | ❑ | ❑ | ❑ | ❑ | ❑ |
| I find my work inspiring. | ❑ | ❑ | ❑ | ❑ | ❑ |
| I am proud of the work I do. | ❑ | ❑ | ❑ | ❑ | ❑ |

| Health locus of control | | | | | | |
| --- | --- | --- | --- | --- | --- | --- |
| Did it happen during the past 6 months, that you went to work while you should have called in sick? | | | | ❑ No  ❑ Yes, sometimes  ❑ Yes, several times | | |
| Please indicate how often each of the following statements applies to you about how you influence your health: | Strongly disagree | Disagree to certain extent | Neither agree nor disagree | | Agree to certain extent | Strongly agree |
| If I fall ill my own behaviour determines how quickly I get better | ❑ | ❑ | ❑ | | ❑ | ❑ |
| If I take good care of myself, I will fall ill less quickly | ❑ | ❑ | ❑ | | ❑ | ❑ |
| What I do is the most important determinant of my health | ❑ | ❑ | ❑ | | ❑ | ❑ |

| Emotional exhaustion | | | | | |
| --- | --- | --- | --- | --- | --- |
| Please indicate how often the following statements apply to you: | Never | A couple of times a year | A couple of times a month | A couple of times a week | Nearly every day |
| I feel mentally exhausted as a result of my work. | ❑ | ❑ | ❑ | ❑ | ❑ |
| I feel gutted at the end of a working day. | ❑ | ❑ | ❑ | ❑ | ❑ |
| I feel tired when I get up in the morning, with the prospect of another working day ahead of me. | ❑ | ❑ | ❑ | ❑ | ❑ |
| A full day’s work represents a heavy burden for me. | ❑ | ❑ | ❑ | ❑ | ❑ |
| I feel ‘burnt out’ by my work. | ❑ | ❑ | ❑ | ❑ | ❑ |

| Fatigue during the past two weeks (CIS) | | | | | | | |
| --- | --- | --- | --- | --- | --- | --- | --- |
| Please indicate how often the following statements apply to how you felt during the past two weeks. The higher the number the more it applied to you | 1  No, not at all | 2 | 3 | 4 | 5 | 6 | 7  Yes, totally |
| I feel tired | ❑ | ❑ | ❑ | ❑ | ❑ | ❑ | ❑ |
| I feel very active | ❑ | ❑ | ❑ | ❑ | ❑ | ❑ | ❑ |
| Thinking requires effort | ❑ | ❑ | ❑ | ❑ | ❑ | ❑ | ❑ |
| Physically I feel exhausted | ❑ | ❑ | ❑ | ❑ | ❑ | ❑ | ❑ |
| I feel like doing all kinds of nice things | ❑ | ❑ | ❑ | ❑ | ❑ | ❑ | ❑ |
| I feel fit | ❑ | ❑ | ❑ | ❑ | ❑ | ❑ | ❑ |
| I do quite a lot within a day | ❑ | ❑ | ❑ | ❑ | ❑ | ❑ | ❑ |
| I feel weak | ❑ | ❑ | ❑ | ❑ | ❑ | ❑ | ❑ |
| I don’t do much during the day | ❑ | ❑ | ❑ | ❑ | ❑ | ❑ | ❑ |
| When I am doing something, I can concentrate quite well | ❑ | ❑ | ❑ | ❑ | ❑ | ❑ | ❑ |
| I can concentrate well | ❑ | ❑ | ❑ | ❑ | ❑ | ❑ | ❑ |
| I feel rested | ❑ | ❑ | ❑ | ❑ | ❑ | ❑ | ❑ |
| I have trouble concentrating | ❑ | ❑ | ❑ | ❑ | ❑ | ❑ | ❑ |
| Physically I feel I am in a bad condition | ❑ | ❑ | ❑ | ❑ | ❑ | ❑ | ❑ |
| I am full of plans | ❑ | ❑ | ❑ | ❑ | ❑ | ❑ | ❑ |
| I get tired very quickly | ❑ | ❑ | ❑ | ❑ | ❑ | ❑ | ❑ |
| I have a low output | ❑ | ❑ | ❑ | ❑ | ❑ | ❑ | ❑ |
| I feel no desire to do anything | ❑ | ❑ | ❑ | ❑ | ❑ | ❑ | ❑ |
| My thoughts easily wander | ❑ | ❑ | ❑ | ❑ | ❑ | ❑ | ❑ |
| Physically I feel in a good shape | ❑ | ❑ | ❑ | ❑ | ❑ | ❑ | ❑ |

| Work | | | |
| --- | --- | --- | --- |
| How many people work at your company | | ❑ 1  ❑ 2-19  ❑ 20-99  ❑ 100-499  ❑ 500 or more | |
| How many hours do you work per week? (only paid work) | | ❑ 0-12  ❑ 13-24  ❑ 25-32  ❑ 32-36  ❑> 36 | |
| Do you have or do you need work adaptations? | | ❑ No  ❑ Yes | |
| Please indicate whether 1) you need one of the following work adaptations and 2) whether you have this work adaptation | 1  Need/want this work adaption | | 2  Have this work adaptation |
| Adjusted working hours | ❑ No ❑ Yes | | ❑ No ❑ Yes |
| Devices/ aids (furniture, tools) | ❑ No ❑ Yes | | ❑ No ❑ Yes |
| Different tasks | ❑ No ❑ Yes | | ❑ No ❑ Yes |
| More job control | ❑ No ❑ Yes | | ❑ No ❑ Yes |
| More support (colleague, supervisor) | ❑ No ❑ Yes | | ❑ No ❑ Yes |
| Other (please specify) …………………. | ❑ No ❑ Yes | | ❑ No ❑ Yes |

| Social support | | | | | |
| --- | --- | --- | --- | --- | --- |
| Please indicate the extent to which you agree with the following statements: | Strongly disagree | Disagree to certain extent | Neither agree nor disagree | Agree to certain extent | Strongly agree |
| My supervisor is concerned about the welfare of the employees | ❑ | ❑ | ❑ | ❑ | ❑ |
| My supervisor pays attention to what you are saying | ❑ | ❑ | ❑ | ❑ | ❑ |
| My supervisor is helpful in getting the job done | ❑ | ❑ | ❑ | ❑ | ❑ |
| My supervisor is successful in getting people to work together | ❑ | ❑ | ❑ | ❑ | ❑ |
| People I work with are competent in doing their jobs | ❑ | ❑ | ❑ | ❑ | ❑ |
| People I work with take a personal interest in me | ❑ | ❑ | ❑ | ❑ | ❑ |
| People I work with are friendly | ❑ | ❑ | ❑ | ❑ | ❑ |
| People I work with are helpful in getting the job done | ❑ | ❑ | ❑ | ❑ | ❑ |

| Physical workload | | | | | |
| --- | --- | --- | --- | --- | --- |
| Please indicate how many hours per day you execute the following tasks at work | never | 1-2 hours | 2-4 hours | 4-6 hours | 6-8 hours |
| Move weights of more than 5 kg | ❑ | ❑ | ❑ | ❑ | ❑ |
| Repetitive movements of arms or hands (not computer) | ❑ | ❑ | ❑ | ❑ | ❑ |
| Long-time same posture | ❑ | ❑ | ❑ | ❑ | ❑ |
| Work behind (computer)screen | ❑ | ❑ | ❑ | ❑ | ❑ |

| Psychosocial work factors | | | | |
| --- | --- | --- | --- | --- |
| Please indicate how often the following statements apply to you in your present position: | Never | Sometimes | Often | Always |
| I have enough time to do my work properly. | ❑ | ❑ | ❑ | ❑ |
| I have to maintain a very high work tempo. | ❑ | ❑ | ❑ | ❑ |
| I have a heavy work load. | ❑ | ❑ | ❑ | ❑ |
| I have to work extra hard. | ❑ | ❑ | ❑ | ❑ |
| My work brings me into emotionally difficult situations. | ❑ | ❑ | ❑ | ❑ |
| My work is emotionally demanding. | ❑ | ❑ | ❑ | ❑ |
| I get emotionally involved in my work. | ❑ | ❑ | ❑ | ❑ |
| I can decide when to perform a task | ❑ | ❑ | ❑ | ❑ |
| My working methods are laid down by others. | ❑ | ❑ | ❑ | ❑ |
| I determine the sequence in which I carry out my activities. | ❑ | ❑ | ❑ | ❑ |
| I decide the approach to be taken to my tasks. | ❑ | ❑ | ❑ | ❑ |

| Home | | | | | |
| --- | --- | --- | --- | --- | --- |
| Please indicate the extent to which you agree with the following statements: | Strongly disagree | Disagree to certain extent | Neither agree nor disagree | Agree to certain extent | Strongly agree |
| I have, next to work, enough free time to spend with my family and friends | ❑ | ❑ | ❑ | ❑ | ❑ |
| I am satisfied with the level of work-home interference | ❑ | ❑ | ❑ | ❑ | ❑ |
| My family/friends are concerned about my welfare | ❑ | ❑ | ❑ | ❑ | ❑ |
| My family/friends are paying attention to what you are saying | ❑ | ❑ | ❑ | ❑ | ❑ |
| My family/friends are always there | ❑ | ❑ | ❑ | ❑ | ❑ |
| My family/friends are paying attention to me as a person | ❑ | ❑ | ❑ | ❑ | ❑ |
| My family/friends support me | ❑ | ❑ | ❑ | ❑ | ❑ |

| Health | |
| --- | --- |
| How do you rate your general health | ❑ excellent  ❑ Very good  ❑ Good  ❑ Fairly poor  ❑ Poor |
| Do you have a long-term disease, disorder or condition? | ❑ No  ❑ Yes, musculoskeletal  ❑ Yes, mental  ❑ Yes, other  ❑ Yes, more than one |
| Does this disease give you long-term health complaints? | ❑ No  ❑ Yes |
| At this moment, do you have health complaints? | ❑ No  ❑ Yes |
| Were you pregnant or did you give birth during the past 6 months? | ❑ No  ❑ Yes |

| Which health complaints do you have at this moment? | Health complaint | Since when  (month-year) | Work is the cause |
| --- | --- | --- | --- |
| Musculoskeletal: shoulders, arms, hands | ❑ | ……-..…. | ❑ |
| Musculoskeletal: Hip, legs, feet | ❑ | ……-..…. | ❑ |
| Musculoskeletal: back | ❑ | ……-..…. | ❑ |
| Mental: overstrain/ burnout | ❑ | ……-..…. | ❑ |
| Mental: depression | ❑ | ……-..…. | ❑ |
| Mental: other mental illness | ❑ | ……-..…. | ❑ |
| Other: migraine/ serious headache | ❑ | ……-..…. | ❑ |
| Other: heart and vascular | ❑ | ……-..…. | ❑ |
| Other: COPD | ❑ | ……-..…. | ❑ |
| Other: gastric/ intestinal | ❑ | ……-..…. | ❑ |
| Other: skin | ❑ | ……-..…. | ❑ |
| Other: life threatening (cancer, aids) | ❑ | ……-..…. | ❑ |
| Other: fatigue, concentration | ❑ | ……-..…. | ❑ |
| Other: flu, cold | ❑ | ……-..…. | ❑ |
| Other: other | ❑ | ……-..…. | ❑ |

| **Health and work performance** | |
| --- | --- |
| Does one or more of your health complaints interfere with your work performance? | ❑ No  ❑ Yes |
| Which health complaint interferes most with your work performance? | ❑Musculoskeletal  ❑ Mental  ❑Other |

| **Sickness absence** | | | | | | |
| --- | --- | --- | --- | --- | --- | --- |
| Did you call in sick between [starting date] and [end date]? | | | ❑ No  ❑ Yes | | | |
| How often did you call in sick between [starting date] and [end date]? | | | …………………… times | | | |
| How many days did you call in sick between [starting date] and [end date]? | | | …………………… days | | | |
| Were you reported sick on [end date]? | | | ❑ No  ❑ Yes, but not 100%  ❑ Yes, 100% | | | |
| Do you receive a disability pension? | | | ❑ No  ❑ Yes, but not 100%  ❑ Yes, 100% | | | |
| For each sickness absence spell the respondent is asked to fill in the starting date, end date and the reason for the absence spell. The reason could be: musculoskeletal health complaints, mental health complaints, flu or cold, other health complaints, conflict at work, private problems, other non-health reasons | | | | | | |
| Absence spell 1 | Start | End | | Reason | % reintegration | Work is cause of absence yes/no |
| Absence spell 2 | Start | End | | Reason | % reintegration | Work is cause of absence  yes/no |
| Absence spell 3 etc. | Start | End | | Reason | % reintegration | Work is cause of absence  yes/no |
| Do you think this list of absence spells is complete? | | | ❑ No  ❑ Yes | | | |

| If no sickness absence was reported during the 6 month study period | | | | | |
| --- | --- | --- | --- | --- | --- |
| How important are or were each of these reasons in preventing sickness absence during the past 6 months | Not important at all | slightly important | Neither important nor unimportant | Fairly important | Very important |
| I had no health complaints | ❑ | ❑ | ❑ | ❑ | ❑ |
| My friends and/or family supported me | ❑ | ❑ | ❑ | ❑ | ❑ |
| I enjoy work/ I am motivated to work/ I do not easily call in sick | ❑ | ❑ | ❑ | ❑ | ❑ |
| There is no work-home conflict | ❑ | ❑ | ❑ | ❑ | ❑ |
| My colleagues supported me | ❑ | ❑ | ❑ | ❑ | ❑ |
| My supervisor supported me | ❑ | ❑ | ❑ | ❑ | ❑ |
| I had sufficient workplace adjustments | ❑ | ❑ | ❑ | ❑ | ❑ |
| My employer paid for medical treatment | ❑ | ❑ | ❑ | ❑ | ❑ |
| I received good medical treatment for my chronic disease/ health complaints | ❑ | ❑ | ❑ | ❑ | ❑ |
| There were no waiting lists so I received treatment without delay | ❑ | ❑ | ❑ | ❑ | ❑ |
| Referral to a health care provider was quick and correct | ❑ | ❑ | ❑ | ❑ | ❑ |
| Different health care providers exchanged the right information (GP, OP, specialist etc.) | ❑ | ❑ | ❑ | ❑ | ❑ |
| Other, please explain …………………………………………. | ❑ | ❑ | ❑ | ❑ | ❑ |

| If sickness absence was reported during the 6 month study period | | | | | |
| --- | --- | --- | --- | --- | --- |
| How important are or were each of these reasons explaining your sickness absence during the past 6 months | Not important at all | slightly important | Neither important nor unimportant | Fairly important | Very important |
| I was too sick to work | ❑ | ❑ | ❑ | ❑ | ❑ |
| My friends and/or family did not support me | ❑ | ❑ | ❑ | ❑ | ❑ |
| I hate work | ❑ | ❑ | ❑ | ❑ | ❑ |
| There is a work-home conflict | ❑ | ❑ | ❑ | ❑ | ❑ |
| My colleagues did not support me | ❑ | ❑ | ❑ | ❑ | ❑ |
| My supervisor did not support me | ❑ | ❑ | ❑ | ❑ | ❑ |
| I had insufficient workplace adjustments | ❑ | ❑ | ❑ | ❑ | ❑ |
| My employer did not pay for medical treatment | ❑ | ❑ | ❑ | ❑ | ❑ |
| I received bad medical treatment for my chronic disease/ health complaints | ❑ | ❑ | ❑ | ❑ | ❑ |
| There were long waiting lists so I received treatment with a delay | ❑ | ❑ | ❑ | ❑ | ❑ |
| Referral to a health care provider was not correct and took too long | ❑ | ❑ | ❑ | ❑ | ❑ |
| Different health care providers did not exchange the right information (GP, OP, specialist etc.) | ❑ | ❑ | ❑ | ❑ | ❑ |
| Other, please explain …………………………………………. | ❑ | ❑ | ❑ | ❑ | ❑ |
| My sickness absence duration could have been shorter or even prevented | ❑ No  ❑ Yes | | | | |

| **Contact with health care providers (by phone or a visit)** | | | | | | | | | | | |
| --- | --- | --- | --- | --- | --- | --- | --- | --- | --- | --- | --- |
| Did you have contact with one of the following health care providers between [starting date] and [end date]? | no | | | | yes | | | | How many times | | |
| GP | ❑ | | | | ❑ | | | | ……… | | |
| OP/specialist on labour (scrollmenu) | ❑ | | | | ❑ | | | | ……… | | |
| Physical therapist | ❑ | | | | ❑ | | | | ……… | | |
| Psychologist/ psychiatrist | ❑ | | | | ❑ | | | | ……… | | |
| Social worker | ❑ | | | | ❑ | | | | ……… | | |
| Medical specialist (scrolmenu with 18 possibilities) | ❑ | | | | ❑ | | | | ……… | | |
| Alternative provider | ❑ | | | | ❑ | | | | ……… | | |
| Dentist | ❑ | | | | ❑ | | | | ……… | | |
| Additional medical testing | ❑ | | | | ❑ | | | | ……… | | |
| Other, please explain ………………………… | ❑ | | | | ❑ | | | | ……… | | |
| For each health care provider and each contact the respondent is asked to fill in the date and the reason. The reason could be: musculoskeletal health complaints, mental health complaints, flu or cold, other health complaints, conflict at work, private problems, other noon-health reasons | | | | | | | | | | | |
| Contact [health care provider] 1 | | | | | | | Date | | | Reason | |
| Contact [health care provider] 2 | | | | | | | Date | | | Reason | |
| Contact [health care provider] 3 etc. | | | | | | | Date | | | Reason | |
| Do you think this list of contacts is complete? | | ❑ No  ❑ Yes | | | | | | | | | |
| Are all contacts with the [health care professional] because of the same reason? | | ❑ No  ❑ Yes | | | | | | | | | |
| Who paid for the treatment? | | ❑ Health insurance covered 100%  ❑ Health insurance covered part of the costs  ❑ Employer paid 100%  ❑ Employer paid part of the costs  ❑ I paid  ❑ Other, please explain ………………………………………….. | | | | | | | | | |
| Who initiated the contact with the [health care provider]? | | ❑ I did  ❑ I received an invitation for an appointment  ❑ The GP referred me  ❑ The OP referred me  ❑ A medical specialist referred me  ❑ Other, please explain ………………………………………….. | | | | | | | | | |
| How many days did you have to wait for your appointment with the [health care provider]? | | …………………… days | | | | | | | | | |
| What is your opinion about the number of days you had to wait for your appointment with the [health care provider]? | | ❑ Not applicable, no waiting time  ❑ Too short  ❑ Fine  ❑ Too long | | | | | | | | | |
| For each [health care provider] | | | | | | | | | | | |
| Please indicate the extent to which you agree with the following statements | | | Strongly disagree | Disagree to certain extent | | Neither agree nor disagree | | Agree to certain extent | | | Strongly agree |
| The [health care provider] is/was the right one to treat my health complaints | | | ❑ | ❑ | | ❑ | | ❑ | | | ❑ |
| The [health care provider’s] treatment had no or not enough effect | | | ❑ | ❑ | | ❑ | | ❑ | | | ❑ |
| The [health care provider] takes me seriously | | | ❑ | ❑ | | ❑ | | ❑ | | | ❑ |
| The [health care provider] understands my health complaints | | | ❑ | ❑ | | ❑ | | ❑ | | | ❑ |
| The [health care provider] contributed considerable to my recovery | | | ❑ | ❑ | | ❑ | | ❑ | | | ❑ |
| The [health care provider] gave medical advice conflicting earlier advise from another health care provider | | | ❑ | ❑ | | ❑ | | ❑ | | | ❑ |
| I am satisfied about the [health care provider] | | | ❑ | ❑ | | ❑ | | ❑ | | | ❑ |
| When no sickness absence | | | | | | | | | | | |
| The [health care provider] contributed considerable to my being able to continue to work | | | ❑ | ❑ | | ❑ | | ❑ | | | ❑ |
| When one or more spells of sickness absence | | | | | | | | | | | |
| The [health care provider] contributed considerable to my (partly) return to work | | | ❑ | ❑ | | ❑ | | ❑ | | | ❑ |

| **Contacts at work** | | | | | | | | | | |
| --- | --- | --- | --- | --- | --- | --- | --- | --- | --- | --- |
| Did you have contact with one of the following people at work about your health complaints between [starting date] and [end date]? | | | no | | | | yes | | Not applicable | |
| Direct supervisor | | | ❑ | | | | ❑ | | ❑ | |
| Boss of direct supervisor | | | ❑ | | | | ❑ | | ❑ | |
| Direct colleague | | | ❑ | | | | ❑ | | ❑ | |
| Someone else, namely …………………………… | | | ❑ | | | | ❑ | | ❑ | |
| For each [work contact] | | | | | | | | | | |
| Who initiated the contact with [work contact]? | | | | | ❑ I did  ❑ The [work contact] did  ❑ Someone else did | | | | | |
|  | Please indicate the extent to which you agree with the following statements | Strongly disagree | | Disagree to certain extent | | Neither agree nor disagree | | Agree to certain extent | | Strongly agree |
|  | The [work contact] take me seriously | ❑ | | ❑ | | ❑ | | ❑ | | ❑ |
|  | The [work contact] understands my health complaints | ❑ | | ❑ | | ❑ | | ❑ | | ❑ |
|  | The [work contact] provides sufficient support | ❑ | | ❑ | | ❑ | | ❑ | | ❑ |
|  | The [work contact] contributed considerable in my recovery | ❑ | | ❑ | | ❑ | | ❑ | | ❑ |
|  | The [work contact] gives advice that is in conflict with earlier advice from a health care provider | ❑ | | ❑ | | ❑ | | ❑ | | ❑ |
|  | I am satisfied with the [work contact] | ❑ | | ❑ | | ❑ | | ❑ | | ❑ |
|  | When no sickness absence | | | | | | | | | |
|  | The [work contact] contributed considerable to me being able to continue to work | ❑ | | ❑ | | ❑ | | ❑ | | ❑ |
|  | When one or more spells of sickness absence | | | | | | | | | |
|  | The [work contact] contributed considerable to my (partly) return to work | ❑ | | ❑ | | ❑ | | ❑ | | ❑ |

| When no health complaints | |
| --- | --- |
| Do you expect to get health complaints during the coming 6 months? | ❑ No  ❑ Yes |
| When health complaints | |
| What do you expect about the seriousness of your health complaints during the coming 6 months? | ❑ Diminish or disappear  ❑ Stay the same  ❑ Increase |
| Everybody | |
| Do you expect to have one or more sickness absence spells during the coming 6 months? | ❑ No  ❑ Yes |

THaNK YOU FOR TAKING THE TIME TO COMPLETE THIS QUESTIONNAIRE.
